# Polydopamine-Assisted Immobilization of Chitosan Brushes on a Textured CoCrMo Alloy to Improve its Tribology and Biocompatibility

**DOI:** 10.3390/ma12183014

**Published:** 2019-09-17

**Authors:** Liguo Qin, Hongjiang Sun, Mahshid Hafezi, Yali Zhang

**Affiliations:** 1 Key Laboratory of Education Ministry for Modern design & Rotary-Bearing system, Xi’an Jiaotong University, Xianning west road, Xi’an 710049, China; sunhongjiang123@stu.xjtu.edu.cn (H.S.); mahshid@stu.xjtu.edu.cn (M.H.); 2 Institute of design science and Basic component, Xi’an Jiaotong University, Xianning west road, Xi’an 710049, China; 3 Key Laboratory of Biomedical Information Engineering of Ministry of Education, Xi’an Jiaotong University, Xianning west road, Xi’an 710049, China

**Keywords:** surface texture, friction, polydopamine, chitosan brushes, biocompatibility

## Abstract

Due to their bioinert and reliable tribological performance, cobalt chromium molybdenum (CoCrMo) alloys have been widely used for articular joint implant applications. However, friction and wear issues are still the main reasons for the failure of implants. As a result, the improvement of the tribological properties and biocompatibility of these alloys is still needed. Thus, surface modification is of great interest for implant manufacturers and for clinical applications. In this study, a strategy combining laser surface texturing and chitosan grafting (mussel inspired) was used to improve the tribological and biocompatible behaviors of CoCrMo. The microstructure and chemical composition were investigated by atomic force microscopy, scanning electron microscopy, and X-ray photoelectron spectroscopy, respectively. The tribological properties were discussed to determine their synergistic effects. To evaluate their biocompatibility, osteoblast cells were cocultured with the modified surface. The results show that there is a distinct synergistic effect between laser surface texturing and polymer brushes for improving tribological behaviors and biocompatibility. The prepared chitosan brushes on a textured surface are a strong mechanism for reducing friction force. The dimples took part in the hydrodynamic lubrication and acted as the container for replenishing the consumed lubricants. These brushes also promote the formation of a local lubricating film. The wear resistance of the chitosan brushes was immensely improved. Further, the worn process was observed, and the mechanism of destruction was demonstrated. Co-culturing with osteoblast cells showed that the texture and grafting have potential applications in enhancing the differentiation and orientation of osteoblast cells.

## 1. Introduction

In recent years, cobalt and its alloys (e.g., the commercial cobalt chromium molybdenum (CoCrMo) alloy) have become well known for their orthopedic and dental applications. For orthopedic treatment, CoCrMo is commonly used in articulating load-bearing implants because of its high performance in wear resistance and corrosion resistance [1,2,3,4,5,6,7]. With aging of the population and an increase in active lifestyles, the demand for all these replacements is increasing [8]. However, revision surgeries are also common. There are several reasons for revision surgeries, such as debris-induced osteolysis, metal ion releasing caused by tribocorrosion, and ascetic loosening [9]. In order to prolong an implant’s life, different surface modifications have been investigated for enhancing the wear resistance properties of the CoCrMo, including thermal oxidation, thin film coatings, surface textures, and nitrogen diffusion [10]. 

Designing and fabricating a microstructured topography called surface texturing is an alternative method and has tremendous technological importance. By introducing microstructures, the surface area can be enhanced. Laser surface texturing (LST) is regarded as one of the most powerful ways to improve the tribological properties of the rubbing surfaces [6,11]. For example, Langhorn produced micro-dimple patterns on a CoCrMo alloy and found that the tribopair wear of polyethylene was reduced by more 50% [12]. Chan used laser ablation on CoCrMo and a greatly enhanced wear resistance was found [13]. Voevodin et al. investigated laser surface texturing of TiCN coatings to permit storage of solid lubricants and results showed that the lifetime of solid lubricants on dimpled surfaces was multifold longer than those on an unmodified surface [14]. In addition, the texture induced on the surface has important applications in the following broad areas of research and development, such as amplifying cell adhesion and proliferation for biomedical applications, enhancing light reflecting behaviors in optical usage, and changing the surface wetting performances for anti-biofouling [15,16,17]. 

The usage of polymer brushes is another way to fabricate substrate surfaces with low friction coefficients. Polymer brushes are prepared from different monomers, which act as a protective coating. Increasing researches have been conducted by this method in recent years. Grafted surfaces have been proven to exhibit superlow friction force in specific environments [18]. The mechanism behind polymer brush lubrication can be attributed to the highly stretched arrangement of its polymer chains. Due to solvation, the generation of an osmotic repulsive force repels the applied load, which can effectively avoid the occurrence of the substrate with direct contact. Thus, a flowing layer is easily formed between the frictional interfaces, which results in ultralow friction when the interface is sheared [19]. Different polymer brushes are also investigated to enhance interfacial properties, such as bioaffinity sensing, specific protein binding, and antibacterial activity [20,21,22,23]. Amongst these properties, chitosan (CS) is one such promising biomaterial because of its high biocompatibility, biodegradability, and antibacterial activities [24]. To conveniently facilitate the grafting of chitosan brushes on substrates, many methods of surface modification have been applied. Qin used a layer-by-layer grafting technique to graft chitosan molecules on Co–Cr–Mo alloys, and the biocompatibility was improved [25]. In order to simplify the grafting process, a polydopamine (PDA)-based surface modification method inspired by marine mussels has gained significant attention, and PDA can be easily used as a strong glue in various environments [26]. Compared to other surface modification technologies, the PDA based grafting method is more convenient to realize [27]. A PDA-modified surface introduces amino and hydroxyl groups, which may keep a relatively long-term stability. Even so, there have been few reports about investigations into PDA-modified chitosan brushes. The preparation of PDA assisted chitosan brushes might contribute interesting knowledge to the field of tribology and biocompatibility. 

Since previous studies have shown that surfaces with texture can enhance load-carrying capacity, and grafted polymer brushes can increase lubricity. A strategy combining texturing and grafting is proposed in the study to prolong the service life of commercial CoCrMo. Tribological properties, combined with a laser textured surface and chitosan brushes, are discussed to determine their synergistic effects. For the fabrication process, CoCrMo was initially laser-textured, followed by covalently immobilizing chitosan brushes onto the textured surface which was with assistance of polydopamine. Then, the wettability and tribological properties were compared and analyzed. Meanwhile, it is also commonly known that the biocompatibility of material for medical applications should be considered first. To provide new insights for medical applications, the differentiation and stained morphologies of the osteoblast MC3T3-E1 cells were investigated to evaluate their biocompatibility.

## 2. Materials and Methods 

The cobalt chromium molybdenum (CoCrMo) alloy (ASTM F75, Steel Material Technology Co., Ltd., Nanjing, China) was used. The samples were cut into dimensions of 12 mm × 6 mm × 2.5 mm. One 12 mm × 6 mm face was polished to a mirror finish by a series of sand grinding papers up to 1600 grit. Then, the samples were ultrasonically cleaned in ethanol and distilled water, following by drying in an atmosphere. The surface roughness (Ra) was measured by a surface profiler (TR-200, Time Group, China), and the Ra was ~80 nm.

**Figure 1:**
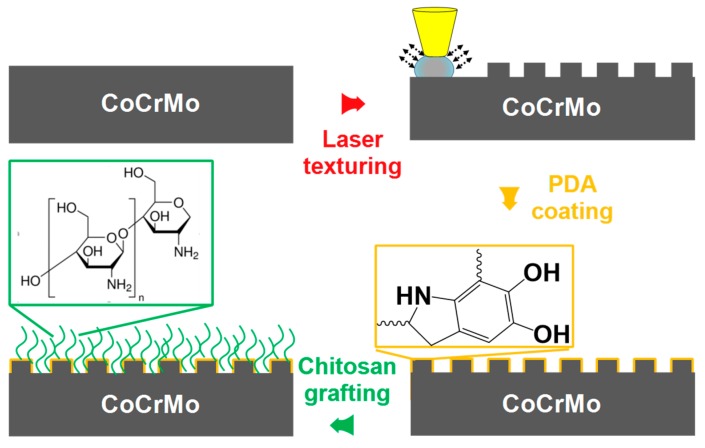
Schematic of polydopamine mediated immobilization for grafting chitosan onto the textured CoCrMo surface.

The density of the texture for lubricated components should be between 5% and 25% to obtain good lubrication performance [6,28]. As shown in Figure 1, a nanosecond laser fiber machine (QC-F20, Qinchuang, China) with a wavelength of 1064 nm was used to introduce the surface texture. The focused laser beam was delivered by a galvanometer scanner and a F-Theta focusing lens, and the laser pulse duration and peak fluency were 200 ns and 20 J/cm^2^, respectively. The dimple radius and pitch between dimples were approximately r = 50 μm and p = 200 μm, respectively. Area densities were calculated to be around 20%. The average output power, laser repetition frequency, and scanning speed were fixed at 20 W, 20 kHz, and 600 mm/s, respectively. After the laser manufacturing process, the substrates were ultrasonically cleaned in ethanol and then rinsed with distilled water and ethanol, respectively. The sample was denoted as Co–T.

The sequential deposition of PDA and chitosan was performed by immersing the substrates into different solutions at room temperature (24 ± 2 °C). Firstly, a tris(hydroxymethyl)aminomethane HCl buffer solution was prepared of concentration 10 mM and the pH was adjusted into 8.5. 0.1 g dopamine was solved in 50 mL solution. Clean and dried samples were immersed for 3 h and kept in dark for the PDA coating. Secondly, PDA-coated samples were washed by distilled water and then were immersed in the solution, which was composed of 1 wt% chitosan (110 kDa, ~75% deacetylated, sigma, USA), 1 wt% acetic acid, and 98 wt% deionized water. The samples and solution were kept static for 12 h. Last, samples were rinsed by distilled water and dried in oven at 37 °C. Both the flat and textured surfaces were used. These surfaces are denoted as Co–S and Co–T–S, respectively.

The morphologies of the samples were observed by field emission scanning electron microscopy (FE-SEM, TESCAN, USA) and 3D laser confocal microscopy (OLS4000, Olympus, Japan). An Innova Digital Nanoscope atomic force microscope (AFM) (Veeco, Plainview, NY, USA) was used to investigate the topographic details. Under tapping mode, a scanning area of 5 μm × 5 μm was performed, and the scanning rate was set to 0.8 Hz Commercial silicon cantilever probes (RTESP, Bruker, Billerica, MA, USA) with a spring constant of 0.35 N/m and a nominal tip radius of 8 nm were selected. The chemical composition of grafting surface was obtained by attenuated total reflectance–Fourier transform infrared spectroscopy (ATR-FTIR) (TENSOR27, Bruker, Karlsruhe, Germany) and ultra X-ray photoelectron spectroscopy (XPS) spectrometer (AXIS-ULTRA DLD, Kratos, UK). The elements of worn surface were measured by an energy-dispersive spectrometer (EDS, OXFORD instruments, Abingdon, UK). The static contact angle (CA) was measured by a video-based contact angle system (JC2000D2A, Powereach, Shanghai, China) at room temperature. The Young–Laplace fitting method was used to calculate the CA value. For every sample, three different areas were tested, and the mean CA was obtained as the apparent CA.

Using a pin-on-disc tribometer (UMT-2, CETR Corporation Ltd., Madison, MI, USA), the tribological performances were investigated via reciprocating sliding. The upper sample was a CoCrMo pin with a diameter of 2 mm. The CoCrMo alloy disc was loaded to reciprocate on a horizontal plane. The reciprocating stroke length was adjusted to 6 mm. For the frictional test the load was set to 2.4 MPa, and the CoCrMo pin was changed into a CoCrMo ball for the acceleration experiment. The ball diameter was 9.5 mm, and the load was 1.2 GPa All tests were carried out under the following conditions; room temperature and relative humidity of ~40%. Lubricants were Bovine Serum Albumin (BSA, Lanji Technology Development Co Ltd., Shanghai, China), and the concentration was 2 mg/mL in NaCl solution. The coefficient of friction (COF) was recorded by the computer. For the observation of morphologies, at least three samples were prepared and three points of each sample were detected. During the wear and frictional test, every data for COF and observation for worn surface were repeated at least twice.

For the biocompatible experiments, MC3T3-E1 cells (a density of 1 × 10^5^ cells/mL) were seeded onto different sample surfaces. The morphologies of the cells were stained and observed under a fluorescence microscope (Ti-s, Nikon, Tokyo, Japan) with different wavelengths. The procedure of cell staining can be found in the previous work [29]. After culturing for 96 h, the cells on the samples were collected, and their RNA was extracted using a TRIzol reagent (Bioteke Corporation, Beijing, China). The sequences of the RT-PCR primers were as same as those of our previous studies [25]. All experiments were performed at least thrice. mRNA of osteoprotegerin (OPG), the receptor activator of NFκB ligand (RANKL) and bone morphogenetic protein 2 (BMP-2) were measured. The fold change in the gene expression was calculated by the 2^−△△Ct^ method, as compared to the flat samples.

## 3. Results

### 3.1. Surface Characterization

**Figure 2:**
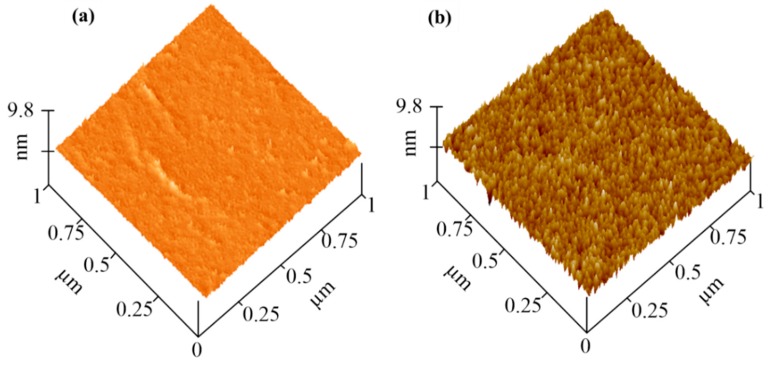
Atomic force microscopy (AFM) morphologies of (**a**) a PDA-modified surface and (**b**) a 12 h CS grafted surface.

The morphology of the CS grafted with flat CoCrMo was characterized through AFM (Figure 2). For PDA modification, a smooth surface was observed, and its root-mean-square (RMS) roughness was approximately 2.35 ± 0.42 nm (Figure 2a). When further immersed into the chitosan solution, the modified surface developed rough, considerable brush peaks (particle-like topography due to the collapse of the attached molecular chains under “dry” conditions were found on the surface [30], and its RMS was increased to 6.61 ± 1.12 nm (Figure 2b). The above characteristics were chosen based on the untextured areas, except in the micro-dimple region. 

**Figure 3:**
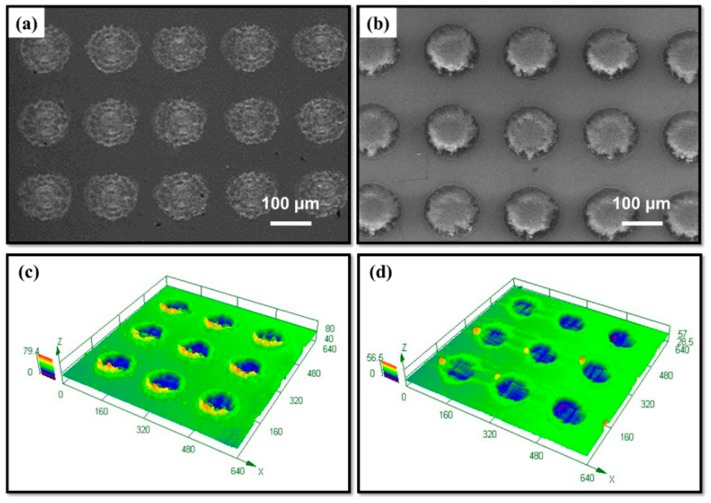
SEM morphologies of the textured surfaces (**a**,**c**) without CS grafting and (**b**,**d**) with CS grafting.

Due to the serious ablation and rough morphology induced by the laser ablation, the micro-dimples could not be accurately characterized by AFM. As shown in Figure 3, the morphologies of the dimples were characterized by SEM and a 3D confocal microscope. After the laser ablation, dimple patterns were observed on the CoCrMo surface. The radius of each dimple was 50 ± 3 μm, and its depth ranged from 13 to 16 μm. Several burrs were found on the edge of the dimples, which were caused by the melting process of the laser (Figure 3c). After grafting CS onto the dimpled surface, there was subtle change of morphologies between the laser textured surface and the ungrafted surface (see Figure 3b,d). This may be due to the grafting thickness of the chitosan in the nanoscale. Micron morphologies of the substrate were unaffected by the nano modification. Meanwhile, the micro-dimples were found to be filled with chitosan polymers. This condition may be related to the difficulty of washing the chitosan molecules that were physically trapped by laser textured dimples. 

**Figure 4:**
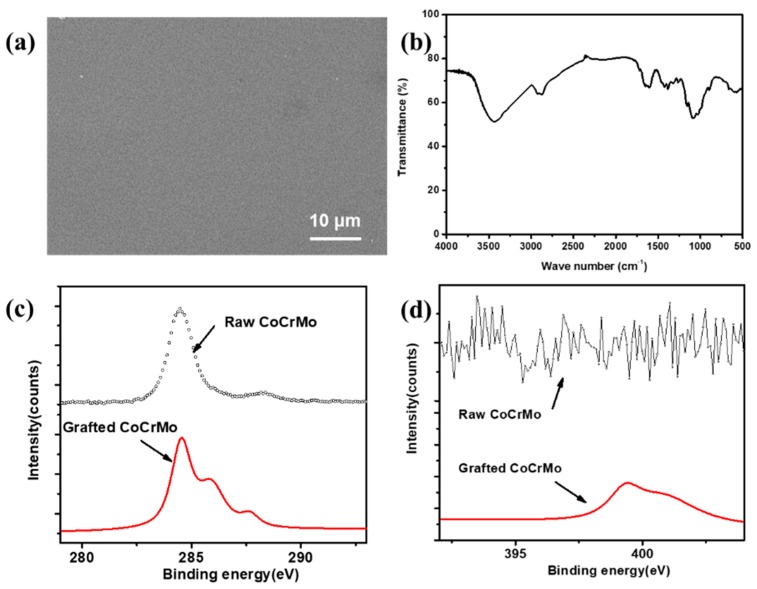
Surfaces grafted with CS brushes: (**a**) SEM morphology, (**b**) FTIR, (**c**) high-resolution spectrum of C1s, and (**d**) the high-resolution spectrum of N1s.

To further confirm the modified surface, SEM, FTIR, and XPS were used to detect and compare the surface compositions of the flat and chitosan grafted CoCrMo surfaces. As shown in Figure 4a, a compact and uniform morphology of the CS coatings was observed. The chemical characteristic peaks of chitosan (Figure 4b) are located at 3412 cm^−1^. The stretching vibration of the hydroxyl group and amide group were found at 1654 and 1584 cm^−1^, which represented the acetylated amino group of chitosan. Figure 4c,d represents the XPS elements’ high concentrations of C and N, respectively. After CS immobilization, the N/C ratio for Co-S was remarkably increased, indicating that the amino chains on the surface also significantly improved. In addition, the high-resolution C1s peak on the surface of the grafted CS sample could be decomposed into three peaks at 284.5 eV, 286.0 eV, and 287.6 eV (Figure 4c), which corresponded to C–C/C=C, C–N/C–O, and C–N=O, due to the condensation reaction between the amino groups and carboxyl groups from the CS brushes. The N1s peak could be decomposed into two peaks at 399.5 eV (–NH– of CS) and 401.5 eV (–CO–NH– and C=N of dopamine) (Figure 4d). These indicated that CS brushes were successfully grafted onto the CoCrMo surface. Meanwhile, PDA was an ideal candidate for grafting chitosan brushes onto the CoCrMo substrate, which normally proves difficult to be modified by general chemical processes.

### 3.2. Surface Wettability

**Figure 5:**
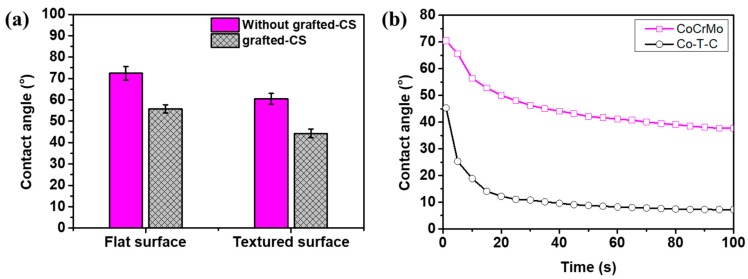
Contact angle in aqueous solution: (**a**) static contact angles and (**b**) the evolution with time increased.

Among various surface properties, surface wetting plays an important role in controlling lubrication. After successfully grafting chitosan onto flat and textured CoCrMo surfaces, the static contact angles (CA) of surfaces with different treatments were measured. As shown in Figure 5, the contact angle of the flat CoCrMo sample was approximately 73.2°, which reduced to 54.1° after grafting Chitosan, indicating the high hydration of chitosan in the aqueous solution. Chitosan exerted an “anti-polyelectrolyte effect”, and the surface can provide good lubrication for the tribopairs under an aqueous solution, which is ideal for cells to be adhered to. For the laser textured CoCrMo surface, the contact angle decreased to 60.2° and to 39.0° with further grafting of chitosan molecules. The results indicate that the immobilization of CS can effectively increase the wetting performance of flat and textured CoCrMo surfaces. To thoroughly investigate the surface wetting behaviors, the contact angle of the flat CoCrMo and chitosan’s grafted textured surface was measured according to the increase in time (Figure 5b). The CA was ~66.6°, which was close to the theoretical calculating results of the Cassie–Baxter model, indicating that the wetting state of Co–T–S was in the Cassie–Baxter state. Murakami et.al modified hexagonal pillared lattices with cycloolefin polymer and similar wetting transition was found [31]. The liquid droplets gradually covered micro-dimples of the textured substrate resulting in a decrease of the apparent CA. This is due to the strong adsorption effect of the chitosan brushes under an aqueous solution. For the purpose of biomedical applications, the bulk and surface properties are the key factors. The interfacial behavior of biomaterials on the surface in the aqueous environments plays a significant role in assessing the biomaterials’ biocompatibility with living tissue. The surface free energy of a material is related to its contact angle, which is directly correlated to its wettability. 

### 3.3. Tribological Behaviors

**Figure 6:**
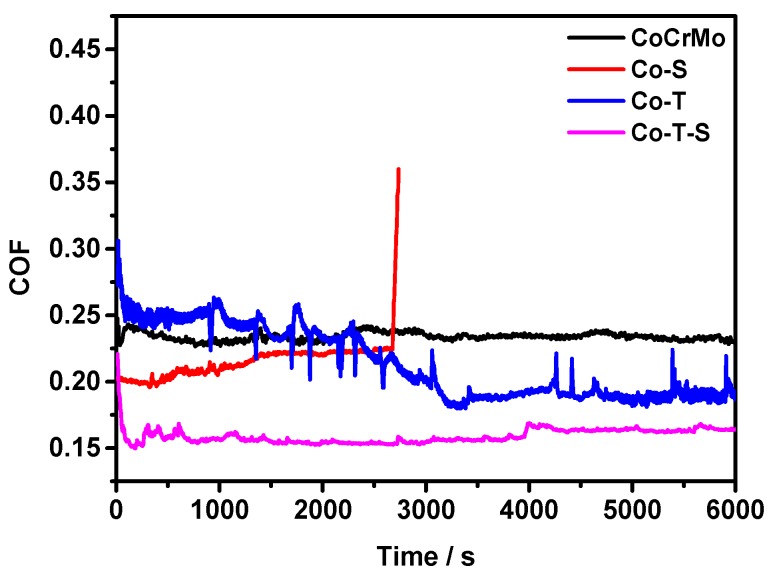
Variations in the friction coefficient of the flat, dimpled, and overgrafted specimens and the overgrafted dimpled specimens as a function of sliding time at a normal load of 2.4 MPa and a frequency of 2 Hz under BSA-lubricated sliding conditions.

Figure 6 shows the friction coefficient of the flat surface, Co-T, Co-S, and Co–T–S as a function of the sliding time under a normal load of 2.4 MPa and a reciprocating frequency of 2 Hz. The results show that the flat specimen had the highest average COF among all the specimens during the entire test period. The laser textured surface gradually decreased to 0.16, which is approximately two-thirds of the flat surface. For the surface grafted with brushes, the COF was lower in the initial state, while the COF sharply increased after the sliding, over a period 2653 s. The COF of CS grafted surface is 0.208, which is approximately 17.4% lower than that of the flat surface. Combining grafting and texturing, the COF is the lowest at ~0.165. The surface texture’s antifriction has been widely identified by numerous studies [6,32]. This quality can be attributed to surface micro-dimples, which decrease the contact area and effectively enhance the fluid-bearing capacity of the surface, thereby lowering the friction force and improving wear resistance under fluid lubrication. In addition, grafting CS brushes can immensely reduce the friction coefficient of the textured surface. The significant antifriction effects of the grafted surface of the polymer brushes have been reported by many researchers, while the lifetime of the coating is limited, as shown in our studies (2653 s). Similar to the storage of lubricants for the textured surface, the thicker layer of the CS in the dimples can transfer onto the sliding area and prolong the service time of the grafted surface. Other studies showed excellent lubrication performance under the aqueous solution [33]. In addition, aqueous environment resulted in a highly stretched conformation of the polymer chains. In the sliding interface, a repulsive osmotic pressure force from the polymer chains was produced, which was opposite to the applied load and effectively enhanced the load-carrying capacity of the surface. A thin fluid lubrication film was easily formed, which resulted in a low surface friction coefficient.

**Figure 7:**
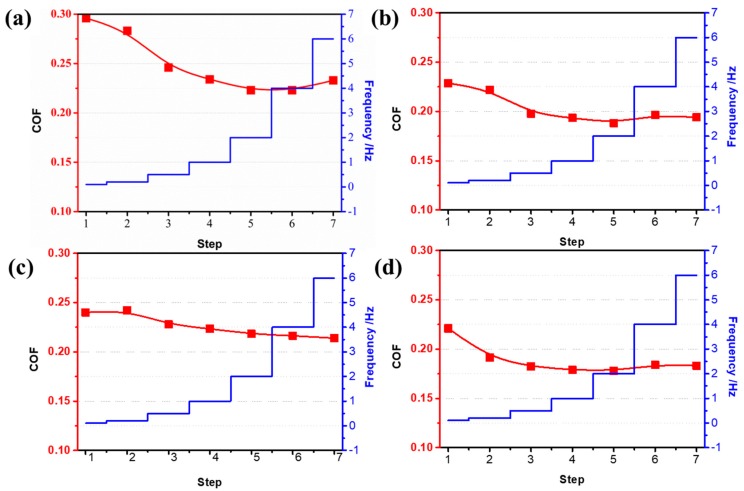
The curve graph of the average COF with different sliding speeds, (**a**) CoCrMo, (**b**) Co-T, (**c**) Co-S, and (**d**) Co–T–S.

To investigate the relationship between the sliding speed and COF, the average coefficient of friction for different samples was performed under different reciprocal sliding conditions. After 30 min, the COF was calculated. As shown in Figure 7, the COF of all frictional tribopairs was decreased when the sliding speed was increased. The COF of the flat CoCrMo was always higher than 0.22. When the surface was textured by the micro-dimples, the friction coefficient was more sensitive to speed than that of the flat surface. According to the elastohydrodynamic lubrication (EHL) theory [34], the load capacity is directly proportional to the value of (L/h)^2^, and the frictional force is proportional to (L/h), where L is the bearing length and h is the minimum thickness of lubrication film. In the initial stage of sliding, Co–T and Co–T–S could achieve these similar EHL conditions. However, the continuous consumption of CS chains resulted in a reduction in the repulsion force derived from osmotic pressure, and this lubrication film could not support a sufficient carrying load under continuous frictional sliding. Therefore, the thickness of lubrication film was decreased. The frictional pairs (pin and disk) were gradually contracted resulting in breakage of chitosan chains and surface wear, which caused the increase of friction force, especially at speeds greater than 2 Hz. When the CS was grafted alone on the CoCrMo surface, the average COF was slightly lower than that of the flat samples, and the COF decreased slightly as the sliding speed increased. From the various speed test, it can be concluded that desired COF is preferred on the surface of CoCrMo by a combination of these types of surface processing technologies. In addition, if the requirement in the tribological properties is not high, one can handle the surfaces using different single technologies. When the speed is low, CS brushes can be deposited on the sliding surfaces, and the abrasion can be reduced. Otherwise, the surface texture is selected to reach a low COF and prevent surface damage. 

**Figure 8:**
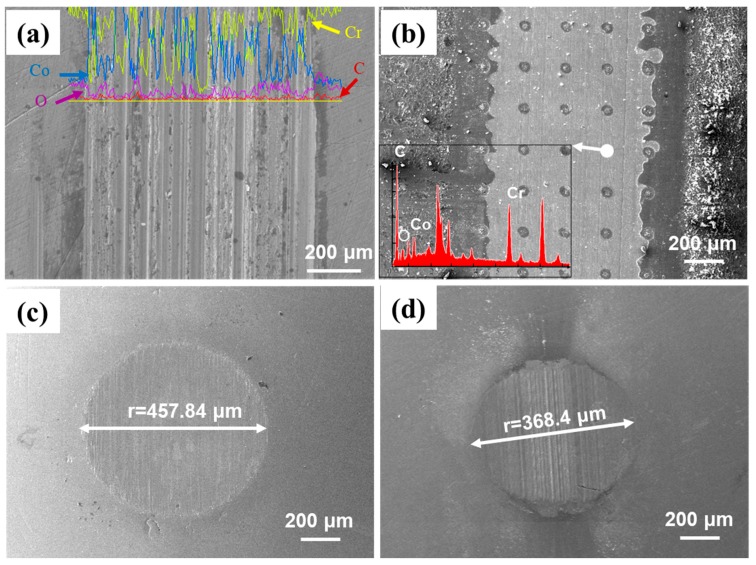
SEM micrographs of the lower disk samples and their corresponding upper ball samples: (**a**,**c**) flat CoCrMo sample and its upper pin and (**b**,**d**) Co–T–S sample and its upper pin. The inserted figures showed the EDS maps of worn surfaces.

To quantify the wear resistance of the Co–T–S sample, accelerating wear experiments were conducted. The pin was changed into a CoCrMo alloy ball whose diameter was 9.5 mm. The sliding time was set as two hours. Accelerated wearing has been carried out to reproduce the strenuous activities in movement of human implants. Figure 8 shows worn micrographs of the lower disk samples and the upper ball samples. As shown in Figure 8a, the worn surface of the flat CoCrMo was severe, and there were a multitude of intensive ploughed grooves. Compared with the flat CoCrMo, several slight ploughs were observed on the contact surfaces of textured samples. As shown in Figure 8b, the worn surface of the disk indicates that the grafted CS chains were severely destroyed. This result indirectly confirms that the failure of the grafted chitosan layer is mainly caused by the accelerating condition. Although the broken signs of the grafted CS brushes could not be observed after such a long period of sliding, the integrity of the circular dimples was obvious. EDS analyses (inserted in Figure 8a,b) further confirmed that rare carbon element was found on the worn surface of flat CoCrMo, whereas a significant detection of carbon was observed for the dimpled area in the worn tract of Co–T–S. Results indicated that the Co–T–S may protect the CoCrMo alloy at least several years. From Figure 8c,d, it can be calculated that the volume loss of the flat CoCrMo disk (with a radius of ~457.84 μm) against the ball was much higher than that of the Co–T–S disk against the ball (with a radius of ~368.4 μm). As mentioned above, the transferred CS from the concave surface to supply the consumption and the highly stretched chitosan chains in the sliding interface are the main reason why the Co–T–S surface could keep long-term stable during the accelerating frictional test.

**Figure 9:**
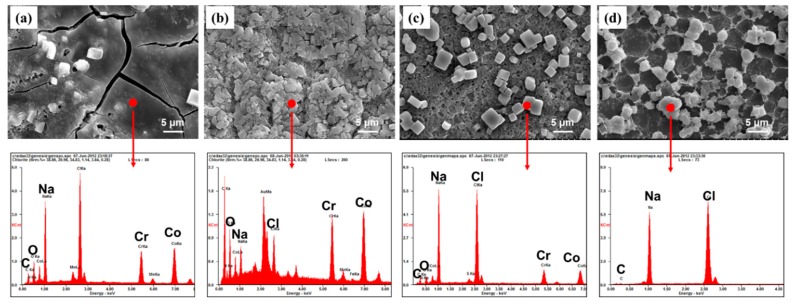
SEM morphologies and EDS of surfaces grafted with CS brushes with different cycles: (**a**) after 100 cycles, (**b**) after 1000 cycles, (**c**) after 5000 cycles, (**d**) after 10,000 cycles.

To further reveal the failure mechanism of the CS grafted surfaces during the wear process, SEM morphologies and the EDS analyses of the surfaces grafted with CS brushes were observed (Figure 9). The wear process of CS brushes grafted on the surface was divided into three stages. Under BSA lubrication, the worn surface was quite smooth, and the adsorbed layers were mainly composed of proteins at the beginning of frictional sliding. The elements contained C, O, Na, and others (Figure 9a,b). In addition to an increase in the rubbing cycles, the adsorbed surface became rough. The content of C and O dramatically decreased, and the Na and Cl largely increased (Figure 9c). This is the second stage of the wearing process. Commonly, this is also the steady state, and its life depends on the rate of consumption. Finally, the thickness of the grafted CS surface gradually decreased (Figure 9d). The matrix of the sample soon began to be exposed. Then, direct contact occurred, and, in the meantime, the COF rose sharply (Figure 6). After 10,000 cycles, the grafted CS was almost worn away. This wear represents the final stage, in which the task of the CS brushes was terminated.

**Figure 10:**
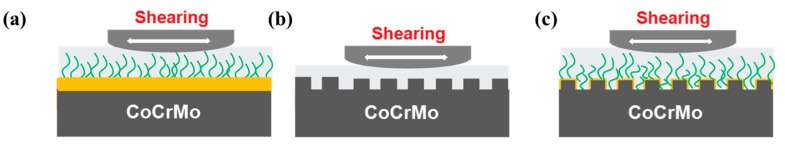
Schematics of the contact mechanics for (**a**) polymer brushes grafted on a flat CoCrMo surface, (**b**) a textured surface, and (**c**) thicker polymer brushes on a textured surface.

Figure 10 shows a schematic of the frictional mechanism of the CS brushes grafted on the flat and textured CoCrMo surfaces, which is based on the obtained experimental results. When the CS brushes were grafted on the flat CoCrMo surface, the CS’s molecular chains were homogenously stretched towards the vertical direction and aligned in the direction as same as each other. Under the aqueous solution, a uniform hydration layer was gradually formed, and thus a low friction of the CS grafted surface was obtained. However, the lifetime of the CS chains was rather short. CS chains were easily destroyed under the alternative shear stress. Meanwhile, the frictional interfaces were separated by an extremely thin hydration layer. Therefore, the service life of the surface with CS grafting alone was transitory. When brushes with long chains were grafted on the textured rough surface, e.g., grafting of CS, the orientation of CS chains were arranged with the substrate’s morphologies [35,36]. In addition, the number of CS chains was reduced on the textured surface due to introduction of numerous circle dimples during the process of laser texturing. Thus, the osmotic pressure’s repulsive force acting was reduced on the friction pair, and the shearing force was weakened accordingly [37]. The weak friction was also observed in Jacob Klein’s investigation when layers of Chitosan were tested [38]. As shown in Figure 10c, although most CS brushes on the micro-dimpled samples showed no direct contact with the frictional tribopair, they still displayed excellent wetting (see Figure 5). Thus, the CS brushes effectively locked lubricants and were kept in the micro-dimples to form a hydrodynamic lubricating film during the sliding process. However, these polymer chains should be longer, as short polymer chains grafted at the bottom cannot influence the lubricating performance on the top surface. Once the polymer chains take part in the surface lubrication effect [39], the formation of lubrication film can prevent the disturbance of the arrangement of grafted CS chains in the region without texturing (i.e., flat area outside the micro-dimples). Meanwhile, CS chains in the concave of micro-dimples showed no direct contact with the sliding pin, which effectively protected the CS chains from consumption. In addition, once the CS brushes outside the dimples were consumed, the stored CS can be supplied onto the rubbing surface. Thus, the CS chains can enhance the lubrication effect between frictional interfaces for a long time. Therefore, Combining with texture can remarkably prolong the service life of CS brushes (see Figure 10).

### 3.4. Biocompatibility

**Figure 11:**
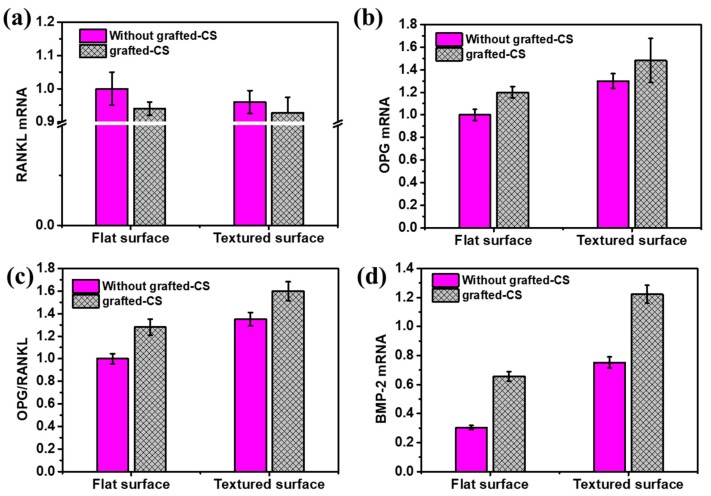
Osteoblast gene expression: (**a**) RANKL mRNA, (**b**) OPG mRNA, (**c**) OPG/RANKL, and (**d**) BMP-2 mRNA.

Osteoblast function is sensitive to the surface topography and surface chemistry of the material. RANKL, OPG, and their ratio and BMP have been proven to play a major role in osteoclastogenesis, and the ratio of OPG to RANKL is an especially critical factor influencing the processes of bone remodeling. The expression of cytokines genes is shown in Figure 11. Compared with the flat CoCrMo samples, the expression of RANKL mRNA for cells cultured on Co–T and Co–T–S was slightly decreased. For OPG mRNA expression, the level was increased for all modified surface. The Co–T–S sample showed the highest expression among the four investigated samples. The ratio of the OPG to the RANKL mRNA expression of MC3T3-E1 cells on the Co–T–S was the highest amongst the four surfaces studied. BMP-2 mRNA was increased to 0.6, 0.75, and 1.2 for Co–S, Co–T, and Co–T–S, respectively. These results suggest that surface chemistry combined with surface topography is a key element in promoting cell proliferation and differentiation [40]. Results indicate that surface textures with CS grafting will have an impact on the indirect regulation of osteoblasts, which will have potential applications in decreasing osteoclastogenesis.

**Figure 12:**
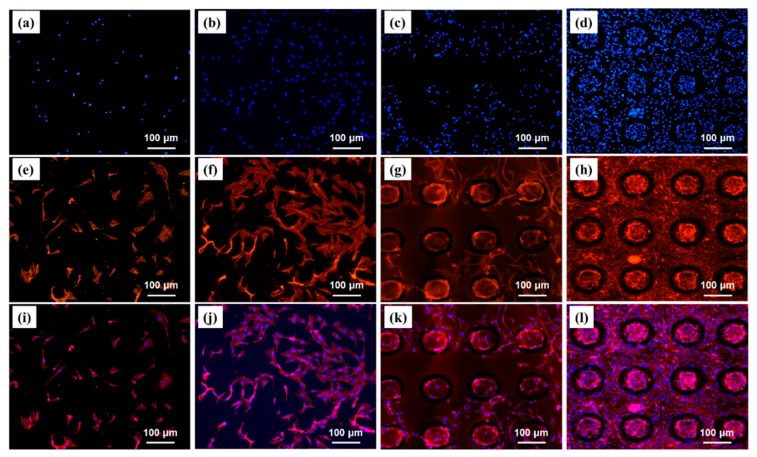
Fluorescence microscope images of cultured MC3T3-E1 cells after seeding for 48 h: (**a**,**e**,**i**) Polished CoCrMo, (**b**,**f**,**j**) Co-S, (**e**,**g**,**k**) Co-T, and (**d**,**h**,**l**) Co–T–S. From top to bottom, nuclear stained cells, actin stained cells, and the two merged cells, respectively.

Figure 12 shows the stained nuclear and actin of cultured MC3T3-E1 cells after seeding for 48 h. For different samples, the cell behaviors were significantly different. The cells were randomly distributed on flat samples, and a lower number of cell attachments was found. For the CS grafted surface, the number of cell attachments were increased, while the distribution of cells was still random. After laser texturing, the cells attached were around the rough regime of the dimples and oriented according to the profile of patterns. Interestingly, with a combination of texturing and grafting, the number of cell attachments and cell orientations were significantly improved (Figure 12d,h,l). The in vitro study of the interactions between cells and substrates (e.g., cocultures) can mimic cellular microenvironments. Substrates modified by textures and coatings were used to promote cell adhesion, proliferation, and differentiation [41]. This is also called contact guidance, a phenomenon where cells align themselves and migrate along the topographies and chemical cues [42]. Contact guidance has been proven to decrease the extent of scar tissue formation and enhance osseointegration. Some researchers have underlined the mechanisms of contact guidance, and several theories have been outlined by experimental research [43,44]. In the study, the response of osteoblast cells to combinations of adhesive cell architecture was investigated at two distinctly dimension scales (a micro-scale with a dimple-like rough structure and nano-scale CS brushes). The cell distribution and orientation were determined by the distribution of micro-dimples, while the grafted CS brushes could serve as good seeding bed and enhance the orientation and proliferation. With a combination of texture and coating, confined cell patterns were realized on a single culture substrate. Recently, it was found that cell differentiation is regulated by an engineered substratum, such as the topography and chemical cues. Homogenous cell-adhesive surfaces with directional topographies was fabricated and oriented cells were found over the entire surfaces [45]. Meanwhile, cell morphologies have been demonstrated to be indicators of the commitment that determines osteoblastic lineage [46]. A thorough knowledge of the interactions between the different surface features and biological responses to biomaterials is further required. This knowledge will facilitate the usage of innovative biomaterials and surfaces in medical applications.

## 4. Conclusions

To immobilize the chitosan brushes on the CoCrMo substrate and produce relatively long-term stability, dimpled surfaces were firstly introduced onto CoCrMo using one step laser ablation, and then bioinspired polydopamine was used. This polydopamine introduced amino and hydroxyl groups that were used to graft chitosan brushes covalently. Using various characterizations, the chitosan brushes were grafted successfully after laser surface texturing. The wettability was improved, and the chitosan grafting successfully formed a lubrication film. The lifespan was significantly improved by grafting the chitosan onto the textured dimples. The MC3T3-E1 cells cultured on the surface were aligned along the pattern direction and the orientation of cells was same as the dimple array. Meanwhile, enhanced osteoblast adhesion and improved cell differentiation were found. Our findings highlight the importance of guidance cues/synergetic effects both in tribology and biocompatibility by laser surface texturing and chitosan grafting. These methods have potential applications in controlling frictional behaviors and tissue organization, which will be of considerable importance for orthopedic implants.
